# Engaging With, Not for: Community-Based Research Towards Inclusive Third Places for Healthy Aging

**DOI:** 10.1093/geroni/igaf122.338

**Published:** 2025-12-31

**Authors:** Mei Lan Fang, Rebecca White, Ryan Woolrych

## Abstract

Despite increasing attention to participatory research, older adults remain underrepresented as active research partners. This symposium presents community-based participatory research (CBPR) projects that go beyond inclusion to foster authentic engagement, challenge ageist stereotypes, and empower marginalized older populations. Each study applies key CBPR principles—equity, inclusivity, empowerment, and co-creation—to innovative participatory methods such as citizen science, co-researcher models, participatory realist review and strategic bricolage, ensuring older adults play a central role in knowledge production in third places. The symposium highlights how community spaces—particularly third places—can serve as catalysts for participation, inclusion, and rights to place. Four papers will illustrate diverse approaches to authentic engagement. White presents the Collective of Older Adult Researchers (COAR) initiative, which addresses tokenistic involvement by embedding intergenerational research training and experiential learning within senior centres, positioning them as research hubs. Fang explores ways to improve collaboration between senior centres, their members, and healthcare partners to develop a community-integrated health model for older adults through strategic cross-sectoral workshops. Stone examines how urban gardening and access to green spaces affect the well-being of older adults, using a realist review co-designed with community partners. Sixsmith shares insights from RomaPlaceAge, a project co-led by Roma community members to address the health and well-being of Roma people aged 40+ in the UK. To conclude, Woolrych, Director of the Urban Institute at Heriot-Watt University, will lead a discussion on the future of participatory research and the critical role of third places in fostering meaningful engagement and social connectivity.

